# Interdisciplinary perspectives on accessing specialty evidence-based treatment for Medicaid-insured adolescents with eating disorders

**DOI:** 10.1186/s40337-024-01124-7

**Published:** 2024-10-22

**Authors:** Peyton Crest, Siena S. Vendlinski, Renee Borges, John Landsverk, Erin C. Accurso

**Affiliations:** 1 Department of Psychiatry and Behavioral Sciences, University of California, San Francisco, CA USA; 2 Rhodes College, Memphis, TN USA; 3 Department of Pediatrics, University of California, San Francisco, CA USA; 4 University of San Francisco, San Francisco, CA USA; 5 Oregon Social Learning Center, Eugene, OR USA; 6 Philip R. Lee Institute for Health Policy Studies, San Francisco, CA USA

**Keywords:** Care coordination, Family-based treatment (FBT), Implementation, Anorexia nervosa, Public insurance, Private insurance, Outpatient treatment, Barriers to care

## Abstract

**Background:**

Family-based treatment (FBT), the leading intervention for adolescents with anorexia nervosa (AN), is severely understudied in outpatient care settings that serve publicly-insured populations. Many individuals with public insurance are lower-income, racially and ethnically diverse, and experience barriers to accessing evidence-based interventions for eating disorders (EDs).

**Methods:**

Semi-structured interviews were conducted with ten interdisciplinary providers who provide specialty care to youth with EDs in an inpatient and/or outpatient medical setting. Interview questions were focused on the interdisciplinary providers’ experiences of caring for individuals with EDs, with a focus on differences in care for those with private insurance compared to public insurance. The interviews took place two years after training in FBT was delivered to mental health providers in San Francisco County, which created opportunities to explore provider perspectives on collaborating with newly-trained mental health providers in the community implementing FBT with publicly-insured youth.

**Results:**

Content analysis converged on three themes: the critical importance of supporting mental health treatment within the context of medical care, complex challenges when helping patients and their families navigate publicly-funded health care systems, and the overall positive impact of the FBT rollout in San Francisco County. Participants emphasized greater confidence in patient outcomes when collaborating with FBT providers and noted discord when working with providers not trained in EDs or FBT. Referral systems, weight-based stigma, and a lack of appropriate services were highlighted as significant barriers to care. To facilitate treatment engagement in publicly-insured populations, participants stressed the importance of clinicians providing psychoeducation and providing services with a high degree of cultural competence. Participants expressed that patients’ ability to access FBT and providers’ ability to collaborate on cases markedly improved following the county training, increasing their sense of efficacy in delivering adequate patient care.

**Conclusions:**

The discussed themes highlight the importance of access to FBT for individuals in underserved communities, which can significantly reduce both provider and patient burden. Despite various barriers to utilizing FBT in publicly-funded settings, clinicians stressed that cultural adaptations increase the implementation of and facilitate family engagement in FBT, which is consistent with previous literature examining evidence-based intervention implementation science.

**Supplementary Information:**

The online version contains supplementary material available at 10.1186/s40337-024-01124-7.

## Abstract (plain language summary)

Publicly-insured individuals often represent lower-income, racially, and ethnically diverse populations who typically lack access to evidence-based treatment for eating disorders (EDs), such as family-based treatment (FBT). Healthcare providers working in a medical setting shared their perspectives on ED care and collaboration with community mental health providers. Participants discussed increased confidence when working with community FBT providers and described common barriers to delivering FBT in publicly-funded settings. To address these barriers, they stressed the importance of cultural competence and patient advocacy. The results of the study suggest that access to FBT providers can reduce patient and provider burden in publicly-funded settings, and highlight the need for improving systems of care to better meet the needs of vulnerable populations with EDs.

**Supplementary Information:**

The online version contains supplementary material available at 10.1186/s40337-024-01124-7.

## Background

Eating disorders (EDs) affect about 20% of the population [1] and carry the second highest mortality rate across psychiatric disorders [2], with significant societal and economic costs [3, 4]. Despite their high mortality and significant costs, the management of EDs in public health systems in the U.S. is not well-described even though Medicaid pays for most mental health treatment in the country. Medicaid is the public health insurance program that provides health care services for low-income individuals in the U.S. for individuals under the age of 65, who represent a racially and ethnically diverse population [5, 6]. Youth insured by Medicaid may experience challenges in accessing EDs care [7, 8], leading to low outpatient therapy use, which may in turn contribute to high rates of hospitalization [9].

EDs present across a wide range of socioeconomic backgrounds, with rates of EDs increasing fastest among individuals with lower socioeconomic status [10, 11]. Given the costs associated with inpatient treatment (e.g., service costs, time missed from work, transportation), access to effective outpatient care is especially important for lower-income populations [5]. However, there are many obstacles to accessing evidence-based outpatient care in community-based settings. For example, providers in publicly-funded settings in the U.S. often lack specialized training in treating EDs and evidence-based interventions [7, 12]. Additionally, stereotypes reinforcing the belief that EDs primarily affect White cisgender females lead to lower ED screening rates among marginalized populations [13]. Delays in identifying EDs and/or initiating treatment compromise patient outcomes and lead to a higher need for more intensive and costly treatment, including inpatient hospitalization [14].

Early intervention is crucial to increase the likelihood of recovery [15–17]. Family-based treatment (FBT) is the leading treatment for adolescents with restrictive EDs, with well-established efficacy [18]. FBT tasks caregivers with the responsibility of renourishing their child, emphasizes taking an agnostic stance towards the pathogenesis of the ED, and externalizes the illness [19]. In addition to improving psychological outcomes, it also minimizes the need for inpatient hospitalization [20]. However, implementation of FBT has largely been constrained to specialist ED settings in national public health care systems [e.g., Australia [21], Canada [22–24], Singapore [25], Finland [26], Denmark [27], or privately-funded settings in the U.S [28, 29]. Differential patient outcomes between specialist and non-specialist care have led to major governmental investments to build out specialist ED services in both the UK [30] and Australia [21]. However, government-funded national health care systems with specialist settings differ significantly in their organization, where evidence-based interventions already represent or readily become the standard of care [21, 27, 30].

The publicly-funded U.S.-based Medicaid system stands in stark contrast to other national health care systems. Medicaid is governed by federal regulations, but states have control over eligibility criteria and allocation of state funds to supplement federal funding, such that Medicaid programs differ significantly across states. California’s Medicaid program (Medi-Cal) serves 5.5 million youth, primarily youth of color (88%, mostly Latinx), one third of whom speak a primary language other than English [31]. Medi-Cal is also unique from other states in that counties have additional independence around the provision of mental health services. Mental health care in California is decentralized, with each county holding responsibility for overseeing mental health service organization, administration, and financing for its members. As a result, variability in organization of mental health services between California counties likely approximates state-to-state level variability. When members require higher level of care specialized ED treatment, counties are responsible for securing a placement through negotiations with private treatment facilities, which is administratively cumbersome and costly [7].

To date, there has only been one published FBT implementation effort within the Medicaid system, which identified important adaptions to address the specific needs of marginalized individuals with EDs receiving care in Medicaid-funded settings. Mental health providers implementing FBT identified several important adaptations to properly attend to cultural considerations, family power dynamics, socioeconomic-related challenges (e.g., food insecurity), and logistical barriers [32]. However, very little is known about how implementation may impact interdisciplinary members of the treatment team, who are critical in the treatment of EDs. In late 2019, San Francisco County supported a county-wide rollout of FBT, in which the senior author provided a two-day FBT training to 25 mental health clinicians across six publicly-funded agencies in San Francisco [33]. We previously examined the perspectives of mental health clinicians in the county who received training and support in implementing FBT [32]. This study builds on the prior work by examining the perspectives of interdisciplinary health care providers who worked alongside the newly trained FBT providers in San Francisco County regarding the implementation process. However, the focus was more on broadly understanding frontline medical providers’ overall experiences in caring for young people with EDs, including the differences in their experiences working with privately-insured versus publicly-insured individuals, as well as navigating connections to mental health.

## Methods

### Context

The initial two-day FBT training for San Francisco County was provided to 25 county mental health providers, most of whom had limited exposure to EDs. The first half-day provided a background on EDs, including training in ED assessment. The remainder of the training was focused on FBT for restrictive EDs, incorporating didactic presentations, role plays, and discussion. While training was consistent with the FBT manual [19], it also incorporated several adaptations designed to improve fit for low-income families, largely allowing for more flexibility to accommodate caregiving, financial, and other needs [17]. Following the training, ten mental health clinicians opted in to weekly FBT consultation for one year, which continued meeting every other week in the second year, during which time eight of them treated ED cases.

In the first two years following the FBT training for San Francisco County, health care providers in the Adolescent and Young Adult Clinic at the University of California, San Francisco (UCSF) placed the most referrals for ED treatment in San Francisco County, and they subsequently collaborated with all front-line mental health providers in San Francisco whose patients received medical ED care at UCSF (i.e., seven of the eight providers who treated ED cases in the two-year consultation period). The Adolescent and Young Adult Medicine Clinic provides primary care to youth up to age 25, as well as specialized medical care to youth with EDs. In the outpatient setting, patients receive medical care, nutrition counseling, and social work support. In the inpatient setting, care is focused on medical stabilization without an inpatient psychiatric unit. In addition to holding responsibility for the medical management of individuals with EDs, this clinic collaborates with mental health providers in the community to support mental health treatment. Integrated psychology/social work staff provide psychoeducation and support to patients with EDs and their families during medical hospitalizations, as well as assisting with treatment planning and referrals in preparation for discharge. In addition to being the largest provider of ED-focused medical care in San Francisco County and their close collaboration with San Francisco County mental health providers, the UCSF Adolescent and Young Adult Clinic has significant reach across patients and counties, having served hundreds of individuals with EDs across 25 counties that year.

### Participants

A purposive sampling approach was used to recruit health care providers across disciplines in the Adolescent and Young Adult Medicine Clinic through targeted, direct outreach via email. We invited one licensed clinical social worker, two inpatient licensed clinical psychologists, and one dietitian to participate, as these four individuals provided most of the interdisciplinary care that supported ongoing medical management. The Medical Director of the UCSF ED Program identified adolescent medicine physicians who cared for the majority of the clinic’s ED patients, six of whom were invited to participate, with a total target sample of approximately ten participants. Except for inpatient psychologists who only provided care to individuals in the inpatient medical stabilization setting, all participants provided care to youth across inpatient and outpatient settings and served youth with both private and public insurance. All providers also had experience in providing FBT-informed care and collaborating with community-based mental health providers with a variety of training backgrounds (i.e., ED specialists and generalists) and across a wide range of therapeutic modalities, including individual and family-based treatment models.

### Procedures

Participants engaged in 60-minute semi-structured individual virtual video-recorded interviews conducted by the senior author, who identifies as a cisgender Latina female. Open-ended interview questions (see Supplemental materials) focused primarily on understanding participants’ experiences in providing care to young people with EDs, with a particular focus on how these experiences may have differed for youth with private versus public insurance; field notes were made following each interview. Topics included their perceptions of FBT effectiveness for this population, as well as their experience collaborating with community-based providers in publicly-funded clinics. The interview also capitalized on the opportunity to learn about participants’ experiences collaborating with FBT providers in San Francisco County in the two years following training. Participants were not re-approached after completion of the interview. The interviewer had working relationships with all participants, and several were aware of her commitment to improve publicly-funded care for EDs and/or her role in the implementation of FBT in San Francisco County. All individuals who were invited to participate provided informed consent, and recruitment ended once thematic saturation was reached at 10 participants. Study procedures were approved by the UCSF IRB.

### Data analysis

The interviews were recorded, transcribed verbatim, and reviewed by both coders and the senior author. After the initial review and familiarization with the data (in video and written form), each transcript was independently coded by two coders using *Dedoose* Version 9.0. The goal of using multiple coders was to explore multiple interpretations of the data and to reflect on the various meanings of the data. A reflexive thematic analysis approach was utilized, with open “data-driven” coding to best capture the data generated from participants [34]. The coders and senior author met frequently to collaboratively and iteratively revise the codebook. Differences in code application were discussed with the ultimate goal of furthering interpretation and data organization to best support theme identification. In addition to inductive analysis, the senior author employed a degree of deductive analysis to ensure that the themes were relevant to the main research questions (i.e., provider perceptions on the rollout of FBT). Transcripts and codes were reviewed and organized by the senior author iteratively to generate initial themes, after which the data were reviewed again, additional codes were added to assist with organizing the data according to themes, and data items were chosen as extracts reflecting of key themes. The methods, results, and discussion in this manuscript are reported in accordance with the COnsolidated criteria for REporting Qualitative research (CORE-Q) Checklist [35].

## Results

Participants (*N* = 10) were mostly women (90%, 10% male) with a mean age of 40.6 (*SD* = 6.92; range: [32,50]). Participants identified their racial and ethnic identity as non-Hispanic White (*n* = 8), Asian (*n* = 1), and Black (*n* = 1). Individual interviews were conducted with medical doctors (*n* = 6, 60%), inpatient psychologists (*n* = 2, 20%), a social worker (10%), and a dietitian (10%). The interviews converged around three main themes. In the first theme, participants emphasized the importance of supporting mental health treatment within the context of medical care. Participants additionally commented on unique mental health needs for Medicaid-insured youth, and they experienced a sense of relief when collaborating with FBT providers, as compared to discord when collaborating with providers not trained in EDs or family-based approaches. Second, participants identified several complex challenges when helping patients and their families navigate publicly-funded health care systems, including frustration with referral systems, limited access to specialty mental health care, and challenges with primary care, all of which contributed to poor patient outcomes and provider burnout. Finally, participants discussed an overall positive experience with the FBT rollout in San Francisco County.

### Supporting mental health treatment

#### Facilitating patient and family engagement in ED treatment

Participants commented on the importance of providing psychoeducation that dispels common misconceptions about EDs and their treatment to facilitate treatment engagement. For example, families who understand EDs as a personal choice had more difficulty “externalizing” the illness (i.e., understanding that the ED is not a choice and outside the young person’s control), making it difficult for caregivers to feel empowered to support their child, both of which are core principles of FBT. Participants reported that families who had received more psychoeducation were often more receptive to FBT principles and had better treatment engagement. Several participants commented on observing a greater need for psychoeducation among families whose children had Medi-Cal insurance, potentially due to lower awareness about EDs and greater levels of mental health stigma.

#### Unique needs for Medicaid-insured youth

All participants identified numerous barriers for Medicaid-insured families participating in FBT, such as caregiver time/resources/capacity limiting their ability to provide meal supervision and emotional support, access to family leave, economic/job security barriers, other dependents requiring support, and food insecurity. Other barriers included providers’ capacity to provide culturally-competent services in the family’s preferred language(s), especially in the context of some negative experiences working with interpreters.

*“It’s fairly common that we’re relying on an interpreter to speak with parents [with public insurance]. It seems like a lot of the nuances are lost*,* literally lost in the translation of the information. [I’m] remembering very clearly speaking with parents of a young person who spoke Cantonese*,* and one parent could understand English and had to correct the interpreter saying*,* ‘This is not a virus.’ So it’s just like*,* ‘Oh my goodness*,* how much inaccurate information is passed along?’ And that has to contribute to ongoing struggles.”* – P05.

They also commented on linguistic and cultural diversity for this population, emphasizing how critical it was to understand families’ cultural context and to deliver culturally appropriate and effective care, and the advantages of having shared social identities. This was especially important in the context of FBT as a family therapy that deals with a culturally-bound topic (i.e., food).

*“If we’re really dreaming big*,* the availability of FBT therapists with different language capabilities would be a huge plus. California is so very diverse*,* and despite our best efforts with translators*,* there are cultural gaps in the ability to deliver care through an interpreter. And then if we’re really*,* really dreaming big… therapists who look like our patients—ethnically*,* racially*,* with similar gender orientations to our patients—so that there is shared understanding of each other… I think that’s really important.”* – P07.

*“All of our snack ideas are very high-dairy*,* very Western snack ideas. I think this is where culturally competent therapists can come into play. For example*,* our Cantonese-speaking therapists would know more what Chinese foods would be more appropriate substitutions within that cultural context… And also understanding—even more nuanced than what is food—is the power dynamic within a traditional Chinese family*,* and being able to read between the lines*,* and challenge them in a way that can be very culturally specific…”* – P01.

Another physician commented how the traditional model of parental empowerment in FBT sometimes runs counter to families’ cultural expectations of healthcare and authority.

*“For example*,* we want to empower the parents to deliver a message that*,* ‘You need to eat this*,*’ or ‘You need to sit down and rest.’ Often*,* they look back to us—as the professionals—and are like*,* ‘No*,* you tell them that. They’ll listen. My child will listen to you because you’re a professional.’ That cultural norm maybe of respecting your elders or professionals is a little different. They may be looking for more of the authority to be conveyed from an outside perspective.”* – P07.

Several participants noted families’ incredible capacity to overcome challenges by leveraging extended social networks (e.g., neighbors, school counselors, and/or family friends to prepare meals, provide meal supervision, and/or provide emotional support and a sense of community) and/or other supports (e.g., Boost Plus prescription, supplementing nutrition by accessing food pantries), as well as the importance of telehealth to increase access for families with long and inflexible work hours. Even for youth involved in multiple systems (e.g., juvenile justice, foster care), participants shared examples of how the system had been creative to identify people who could support renourishment within an FBT-aligned framework. While traditional FBT was not always feasible or appropriate, there was agreement amongst providers that FBT was still the preferred primary treatment available and that its tenets were applicable to families facing multilevel barriers to treatment engagement, even if adaptations were required. Indeed, all participants endorsed their belief that FBT could be as effective for patients insured by Medi-Cal as those with private insurance.

*“I have found that Medicaid families are actually much more willing to start the FBT process. When that recommendation comes down and they’re connected with a therapist*,* it’s game on. They’re willing to do whatever they can to support the young person and follow the letter of the law—assuming complete control of nutrition and the degree of weight restoration that we’re expecting.”* – P07.

*“There hasn’t been any difference whatsoever in the families’ capacity to do FBT. In some cases*,* certainly*,* there are more limited resources*,* in terms of time*,* money*,* caregivers*,* if you’re working with a family who needs to be working full time*,* and can’t take time away. They don’t have a salaried job. They don’t have paid family leave. They don’t have another partner who could step in. But the families that I’ve worked with… have all been incredibly motivated to overcome any of those barriers*,* and to think creatively about how to make it work….”* – P06.

#### Relief when collaborating with providers delivering FBT

Physicians expressed feeling more confident about patients’ prognosis when they were receiving FBT, and they were better able to stay within their scope of practice by redirecting the family to discuss certain queries in therapy. Similarly, the need for nutrition counseling was often minimal, in contrast to families not in FBT whose parents were not empowered and often struggled to support adequate nutrition. One physician remarked on the value of collaborating with FBT providers:

*“When one of my patients is connected with a true gold standard FBT therapist*,* there is just a huge subconscious sigh of relief because I know that I have a partner in treatment—a true partner in crime in terms of really reiterating the same medical goals*,* with weight restoration being important for medical and psychological recovery. I have a partner in determining the little nitty-gritty things that patients will call and email about. ‘My kid wants to start lacrosse practice*,*’ or ‘We’re weight restored. Are three snacks really still necessary?’ …it’s impossible for me to be really thoughtful and triage every single one of those little questions. When there’s an FBT therapist seeing the family weekly*,* really understanding the culture of the family who can help them navigate those conversations*,* it lets me—as the medical provider—focus on the bigger picture*,* which… is important for me to deliver really good care.”* – P07.

Despite feeling strongly that FBT is the best treatment option for many families, one inpatient mental health provider noted that they might recommend other treatment options if those were more readily available at the time of discharge given long waitlists and limited access to FBT in the community, regardless of insurance status.

#### Discord when collaborating with providers not trained in EDs or family-based approaches

Medical providers often noted a disconnect when collaborating with non-FBT mental health providers. This gap was so significant that when individuals were unable to access FBT, one physician felt conflicted about whether some support was better than none in light of having witnessed “harm done by a therapist who has no eating disorder expertise” (P07). Similarly, one inpatient psychologist commented on how support services (e.g., wraparound) could sometimes undermine treatment given providers’ lack of understanding of ED treatment. Providing families with evidence-based guidance while not undermining the patient’s therapist was “a very complicated and time-intensive process” (P10), required more time to collaborate with the therapists, and often left families feeling “paralyzed in terms of conflicting advice” (P07). Physicians also reported scheduling more frequent follow-up appointments with these patients and spending additional time with them during visits (e.g., providing more ED-related psychoeducation, providing mental health counseling, managing family conflict), even though some of these activities were outside of the physicians’ scope and interfered with their capacity to stay within 30-minute appointments.

*“So a family is admitted to the hospital. They get some education [from inpatient mental health team] about what the eating disorder is*,* and the general approach*,* and then they go home*,* and of course*,* the first week is really*,* really hard. There’s tons of pushback. They don’t have an FBT provider to work on that with*,* and so they’re like*,* ‘Well*,* I don’t know. I can’t get her to eat. I can’t force her to eat. What do I say when she says*,* ‘No*,* I’m not going to eat that; you’re making me fat.’?… When they have [an FBT] provider*,* I’d be like*,* ‘This is a really good thing to bring up with your therapist…’ but when there’s not that person*,* then I feel like I have to do more of that counseling and education*,* which again*,* I’m not particularly trained to do.”* – P04.

*“When a patient doesn’t have an FBT therapist*,* there’s an insurmountable amount of information to get through in the context of a 30-minute appointment. I do the best I can… but ultimately*,* to have that partner of an FBT therapist is the best case scenario.”* – P07.

### Challenges with publicly-funded health care

#### Frustration with referral systems

Navigating county systems and resources was complex, especially for certain counties. One physician commented that patients or families may not know how to effectively advocate for the services they need, placing additional burden on the medical providers to provide them with “a lot of coaching” (P04) on what to ask for when calling the county to request services. Providers stressed that having a centralized referral process would make it easier to navigate the referral process, rather than dealing with different processes for each county behavioral health systems and “layer upon layer of red tape” (P06). Several participants who had more recently joined the team relied heavily on colleagues’ institutional knowledge to help them navigate these systems.

*“Just when I think I understand it*,* something new comes up. So*,* it’s very county-dependent*,* and as a provider*,* it’s like you’re stepping into a new state and learning the state system…. It’s kind of a crapshoot whether folks are able to get connected…. So*,* there’s the system and the proper channels*,* but then there’s also having enough experience to know ‘wink-wink’ behind the scenes—who is the person that can actually get this done.”* – P07.

#### Limited access to specialized publicly-funded outpatient mental health care

The Affordable Care Act (ACA) in 2014 established mental health parity by requiring health care coverage for EDs. Prior to that, counties had provided treatment for some mental health diagnoses (e.g., anxiety, depression) but not EDs. However, even years after the ACA was passed, a social worker commented on how some counties had failed to incorporate ED treatment into their systems, leaving the medical team with the responsibility to advocate for their patient to receive appropriate services through the county.

*“[Insurance] is one of the first things I look at when I’m doing a new intake because that changes my behavioral health recommendations and how I streamline and triage folks…. In terms of connecting them with gold standard FBT*,* …it requires a lot of advocacy… FBT therapists are extremely limited through the county behavioral health system…. We’re at the mercy of the system*,* which feels unfair. There’s only so much advocacy the clinical staff can do and the family can do.”* – P07.

*“Even after like 2016*,* 2017*,* when I would call [the counties]*,* they would say to me*,* ‘It’s not a thing. We don’t treat eating disorders.’ And I would say*,* ‘Actually*,* the State says you do.’”* – P03.

*“The county wasn’t responding initially [to our team’s request for services]. They weren’t acknowledging the referral. Then they were like*,* ‘What’s an eating disorder? Oh*,* we don’t have those in our county.’ I’m like*,* ‘You do. You just don’t know how to treat them.’ There was a lack of knowledge and awareness [about EDs]*,* and there’s certainly no FBT-trained clinician available to any of the people in that county.”* – P05.

Even in counties with specialized ED treatment, access was poor due to the low number of providers with ED training and generally limited clinical capacity. Participants frequently highlighted their role as advocates for underserved patients to access appropriate care, especially when treating those with poverty-related stress and challenges navigating care systems. Participants also commented on the level of advocacy needed to mobilize multiple systems (e.g., mental health, child welfare, school district) to deliver the required care.

*“It’s been a really big challenge to advocate for a lot of our patients who have been admitted*,* like 20 times*,* and really need [a higher level of care]. We’re spinning our wheels*,* and readmitting them and readmitting them*,* and they really don’t get the care they need. It’s almost like failure of outpatient therapy*,* but they really never got the outpatient therapy. So*,* then those folks really need higher level of care that they maybe wouldn’t have needed had they just had outpatient therapy.”* – P04.

*“The county wasn’t going to treat the eating disorder…. She’d already been admitted to the hospital 4 or 5 times…. I was so frustrated and so angry… that I wrote [county behavioral health] a letter and I laid out… what the law says about needing to treat them. It turns out that when you write a letter like that*,* the lawyers at County Behavioral Health get concerned…. They authorized a higher level of care and ended up sending that young person to a treatment program.”* – P03.

Many providers also reported a sense of burden and injustice in having to cope with the inequity of some patients not having access appropriate ED care.

*“Identifying [EDs] is always important*,* but if we identify and have nowhere to put them*,* that doesn’t do a whole lot…. If we could have a hub of trained clinicians—especially now that telehealth is more normative—that are able to see people across the state*,* so that it’s not like*,* ‘Sorry*,* you live in this county*,* and that sucks. That’s unfortunate for you. Too bad you don’t live in San Francisco County*,* where we’ve got some good resources for you.”* – P05.

#### Prevalence of weight-based stigma amidst low ED awareness in medical settings

Providers identified that many physicians serving Medicaid-insured populations lacked general training in EDs, likely due in part to misconceptions about which patients are affected by EDs (e.g., thin, White). As a result of poorer screening and diagnosis, Medicaid-insured patients often presented with more long-standing or severe symptoms, as well as heightened family mistrust of the healthcare system that had failed their child. Screening and diagnosis were impacted by weight-based stigma, cultural factors, and language differences, especially with publicly-insured and minoritized populations.

*“Many more of the patients I’ve encountered with Medi-Cal have had prior physicians with a lot of weight-based stigma. I was working with a Spanish language interpreter*,* and we reflected on this together*,* where the parent was told by a physician*,* ‘You should take dieting tips from your kid.’ …not recognizing that an eating disorder was serious. A lot of dismissal of parents’ concern.”* – P07.

*“A lot of times*,* our families are working with pediatricians who have no idea— who know nothing about eating disorders*,* don’t understand FBT—and provide information that’s actually really problematic… and harmful*,* even. There have been several non-English-speaking families with Medi-Cal*,* who had been raising the alarm bell with their physician*,* and the physician was saying*,* ‘You know*,* your child was overweight previously. They’re fine.’ In one case*,* 100 pounds of weight loss*,* to the point where the kid was in severe refeeding when she came in. Mom had advocated with different physicians. Finally*,* a cardiologist said*,* ‘No*,* you need to take her to the hospital.’ The mom was like*,* ‘No one was listening to me.’ Those cases have been really heartbreaking.”* – P06.

*“Parents may have been telling a provider*,* ‘There’s something wrong with my kid; they should be eating more*,*’ and the provider has minimized those concerns… I’m more likely to hear from Medi-Cal families—‘I really tried to get my child help*,* and the medical providers dismissed me.’”* – P02.

In some instances, ED symptoms were even promoted by primary care physicians who encouraged caloric restriction and excessive exercise. A dietitian commented on the systemic rhetoric that weight is a proxy of health, and how challenging weight stigma must occur on both provider and societal levels to create meaningful change in the way EDs are viewed.

*“We get kids that come in*,* and they were told actively that they needed to lose weight—praised for not eating enough and overexercising. And I think physicians and schools and people in general need to be educated on weight stigma…. I think we’ve gotten so used to thinking of health—at least in the healthcare field—as primarily being something physical*,* and something that you can measure by just looking at somebody’s weight…. I think it’s going to require a real shift in the way people view body image and healthy nutrition.”* – P10.

As a result of these experiences, physicians commented on the need for ED training to increase the availability and adequacy of care for patients with EDs, given that specialty services would never be able to meet the clinical demand for ED services.

*“Being able to comfortably manage patients medically who are struggling with eating disorders is not something that we can expect most trainees to be able to do. And so*,* I think that’s a fault in the medical education system.”* – P09.

#### Case management intensity and poor patient outcomes leading to increased provider burnout

Without early access to FBT (and sometimes not even any therapy), many patients became trapped in a revolving door of hospital readmissions and ultimately required a higher level of care, which posed additional economic and emotional strain on families. One of the inpatient psychologists commented on the strain and hopelessness in the family of an adolescent with repeated hospitalizations.

*“The family was feeling really defeated by the process*,* unsupported*,* confused of why it was taking so long [to access mental health services]. And then the father and sister—with mom and patient gone for basically six months [due to hospitalization]—were incredibly frustrated and feeling distant. And the sister was saying incredibly hurtful things like*,* ‘I wish you would die*,*’ or ‘You should just move out and be homeless.’ And then*,* the patient started saying things like that because her eating disorder was raging and getting stronger*,* …and she became hopeless that anything could change.”* – P07.

Patients and their families feeling defeated also contributed to provider burnout. Providers reported feeling frustrated, discouraged, and at times hopeless, especially for young patients without access to FBT. Several participants referenced the intimacy and vulnerability of their relationships with patients, their emotional investment in patients’ well-being, and the meaning they derive from playing a role in their recovery. However, doing such difficult work in the absence of adequate support from the rest of the health care system was disheartening.

*“It’s really draining and extends well beyond the work day. It’s demoralizing. It just feels like*,* ‘What am I even doing?’ I’m not effective. Here’s this person [in the hospital] yet again. Like*,* is there a point to this?”* – P07.

*“I feel like there’s really nothing to offer [patients with Medi-Cal insurance]*,* unless it’s offering them hospitalization. Sometimes I feel like my medical check-ins are useless because they don’t have any therapy support. They don’t have any ability to implement [treatment recommendations] in a meaningful way.”* – P02.

*“I’m deep in hell here. There’s counties where literally*,* every time the young person has a medical follow up*,* regardless of the interval*,* they’re readmitted—where these young people are unstable within three days and requiring lengthy re-admissions. There’s many young people from other counties we’ve gotten to know incredibly well… because they’re readmitted over and over and over and feeling defeated and hopeless.”* – P05.

This posed an additional burden on providers to engage in a lot of non-billable care coordination. Lack of outpatient mental health support also directly impacted physician’s clinical care. For example, if physicians were concerned about the family’s capacity to manage nutrition at home prior to admission to a higher level of care program or in the absence of appropriate linkage to outpatient mental health treatment, they would keep patients in the hospital a bit longer. In addition, providers commented on how their role needed to shift to provide “crisis management” or other additional support to patients and their families in the absence of mental health treatment.

*“With our families with Medi-Cal*,* there are many more meetings and care coordination meetings…trying to get on the same page*,* making sure everyone has shared information*,* shared goals*,* is working together…. It’s a lot of additional meaningful time*,* but that is non-billable for us and above and beyond what we would normally do.” – P07*.

*“I have to provide quite a bit of counseling*,* and I do sometimes struggle with staying in my lane…. If they have no mental health care support*,* I don’t know where else they’re going to hear it. Hopefully from our doctors*,* but if they’re struggling with nutrition and they don’t have that mental health piece…*,* then automatically a lot of the questions*,* conflict [comes up] pretty regularly [in our visits]…. You find yourself stepping into a therapy role helping to manage conflict*,* externalizing [the illness]*,* and reframing*,* and helping the family to shift their perspective on things. You can provide all the nutrition counseling in the world*,* but if they’re having such a hard time with emotions and behaviors*,* implementing that plan is not going to go anywhere.”* – P10.

### Positive impact of FBT implementation on care collaboration

In preparation for the FBT rollout, San Francisco County developed a streamlined referral pathway. Physicians and the social worker expressed satisfaction with being able to directly place FBT referrals, as well as having access to a point person who was coordinating referrals and had expertise to consult on cases, which facilitated a prompt and reliable connection with outpatient FBT services.

*“If someone in San Francisco County with Medi-Cal is admitted [to the hospital]*,* it’s like almost a celebration*,* woo-hoo*,* we have a great resource. There’s a solid program with trained clinicians and a very clear referral pathway. That feels like a big sigh of relief*,* and also knowing that the ball is passed… and feeling less ongoing ownership [of making sure the patient connects to eating disorder treatment].”* – P05.

Physicians also reported high satisfaction with the collaboration on cases—using a shared language, which increased providers’ sense of efficacy and reduced their burnout.

*“I’ve shared different patients with a few of [the FBT clinicians in San Francisco County]*,* and …most of the time*,* it’s been really collaborative. I feel like they make pretty good instinctual decisions. …Especially patients that I don’t see every week*,* I really rely on them for how they feel like [patients] are doing from a psychological cognitions perspective*,* but then [if they’re doing something that might not be typical]*,* to say ‘Oh*,* this is usually our approach. What would you think?’ and being able to collaborate together. I feel like … they’re still kind of new to it*,* so they come with that openness and that willingness to collaborate and create plans together.”* – P08.

Participants also appreciated access to providers within San Francisco who could accommodate families’ cultural and language needs, given attention to training providers who could provide therapy in a variety of languages.

*“Recently*,* I had an inpatient whose parents are Cantonese speaking. The patient is bilingual*,* and we were able to get a patient into FBT with a Cantonese-speaking provider through the county within three weeks. I was floored. I was like*,* ‘Yes*,* this is great!’”* – P08.

However, several participants expressed concern that the mental health clinicians who sought out training were unique. For example, one participant noted that these providers were “more highly motivated and professionally inclined to seek out opportunities for advancement than the average clinician” (P02), and two others expressed concerns that the newly trained providers would end up having larger and more complex caseloads, potentially increasing risk for provider burnout.

*“…it takes a special therapist to sign-up*,* because if you’re working in this system and you sign-up for [a training like this]*,* you will then see more patients. You will then increase the work and see more patients with the thing that you signed-up to be trained in. So*,* the system doesn’t necessarily reward providers that seek out seeing more difficult patients.”* – P07.

*“The very seasoned clinicians…that have worked in the system for 20-plus years*,* they know how to survive in the [public health care] system. How you survive in the system is you have boundaries*,* and you protect yourself from burnout*,* because the system… doesn’t protect you…. So*,* if you’re in a system where clinicians are at a risk of burning out at an outpatient level of care*,* they’re not gonna want to sign-up to see more acute patients*,* and [EDs] are a very difficult population.”* – P01.

## Discussion

These qualitative findings highlight the perceived effectiveness of FBT for youth with EDs across social identities and socioeconomic levels. Given the paucity of data on the feasibility and effectiveness of FBT for minoritized youth from diverse racial and ethnic or lower-income groups and/or FBT implemented in publicly-funded settings, this study offers a unique contribution in that it highlights the importance of access to FBT, especially for young people in underserved communities. Indeed, medical ED expert clinicians attributed poor outcomes (including repeated hospitalizations) to a lack of access to evidence-based mental health care. As a result of this gap, medical providers provided more case management, emotional support, and counseling to patients and their parents, as well as increased coordination with other providers, leading to increased provider burden and burnout. Participants also commented on the prevalence of weight-based stigma and poor ED awareness in medical settings for Medicaid-insured youth, leading to an exacerbation of symptoms and delayed diagnosis, which is aligned with prior findings [36, 37] and could lead to poorer health outcomes [38].

Providers generally reported a positive experience with the FBT implementation in San Francisco County, emphasizing that having access to trained (albeit novice) FBT providers significantly reduced both patient and provider burden. Providers underlined that having “common language,” shared treatment goals, and better-defined treatment team roles when collaborating with FBT providers allowed medical providers to focus on care falling within boundaries of their profession. They also commented on the importance of linguistic and cultural competence to adequately serve this population. Even so, there were several barriers to treatment engagement for under-resourced families, including the financial burden of providing sufficient nutrition, limited time to prepare and supervise meals/snacks, and mental health stigma. These results align with previous literature that underscore time, geographical barriers, and costs as significant barriers to accessing FBT [12], and emphasize the importance of flexibility and leveraging both families’ strengths and resources when implementing FBT with those facing socioeconomic barriers.

This study was conducted with a sample of providers within a specialty EDs program, situated within an academic medical center. The providers had a wide range and depth of experience working with patients with EDs across many distinct county mental health service systems. While providers perceived the implementation effort within San Francisco County as highly successful, the care provided by their team also greatly facilitated positive outcomes, as the implementation of FBT would have been more challenging if community-based providers had been collaborating with medical providers not well-versed in EDs.

## Conclusions

Overall, the findings of this study provide important insights on implementation of FBT for minority populations in under-resourced clinical settings. The results of the study are consistent with previous literature detailing common barriers to care faced in such populations and complement our prior research on mental health clinicians’ identification of necessary cultural adaptations [32]. These data also suggest that there may be large-scale financial implications if increased access to evidence-based outpatient care can prevent or reduce reliance on higher levels of care (e.g., ED programs or inpatient hospitalizations). However, future research is needed to understand the extent to which training and implementation efforts in the outpatient context may improve lead to lower service utilization costs. This study fills a gap in knowledge about implementation facilitators and barriers for FBT, especially those pertinent to publicly-funded systems given that FBT has been assessed primarily in academic medical centers [39], without attention to effectiveness in usual care settings and with marginalized populations [5, 13]. Future research can leverage these findings to facilitate implementation of evidence-based interventions in publicly-funded settings, including organizational-level and individual-level implementation strategies.

## Electronic supplementary material

Below is the link to the electronic supplementary material.

Supplementary Material 1

## Data Availability

The datasets used and analyzed during the current study are available from the corresponding author on reasonable request.

## Abbreviations

AN: Anorexia Nervosa
ED(s): Eating disorder(s)
FBT: Family-based Therapy
UCSF: University of California San Francisco
CORE-Q: COnsolidated criteria for REporting Qualitative research
ACA: Affordable Care Act
