# Correction: Interdisciplinary perspectives on accessing specialty evidence-based treatment for Medicaid-insured adolescents with eating disorders

**DOI:** 10.1186/s40337-024-01144-3

**Published:** 2024-11-14

**Authors:** Peyton Crest, Siena S. Vendlinski, Renee Borges, John Landsverk, Erin C. Accurso

**Affiliations:** 1 Department of Psychiatry and Behavioral Sciences, University of California, San Francisco, CA USA; 2 Rhodes College, Memphis, TN USA; 3 Department of Pediatrics, University of California, San Francisco, CA USA; 4 University of San Francisco, San Francisco, CA USA; 5 Oregon Social Learning Center, Eugene, OR USA; 6 Department of Psychiatry and Behavioral Sciences, University of California, San Francisco, CA USA; 7 Philip R. Lee Institute for Health Policy Studies, San Francisco, CA USA

**Correction: Crest et al. Journal of Eating Disorders (2024) 12:167** 10.1186/s40337-024-01124-7

Following the publication of the original article, the authors reported that the “Availability of data and materials” and “Authors’ contributions” sections were not the updated version.

The correct sections should have read as follows:

**Author contributions**

All authors listed have made a substantial, direct and intellectual contribution to the work, and approved it for publication. ECA conceptualized the study with JL. PC, RB, and ECA were involved in the coding of the data, with PC and RB as the primary coders. PC, SV, and ECA analyzed the data and drafted the manuscript. All authors critically revised and approved the final manuscript.

**Availability of data and materials**

The datasets used and analyzed during the current study are available from the corresponding author on reasonable request.

The original article has been updated.

