# Supplementary material for: Interdisciplinary perspectives on accessing specialty evidence-based treatment for Medicaid-insured adolescents with eating disorders
Source: J Eat Disord. 2024 Oct 22;12:167. doi: 10.1186/s40337-024-01124-7 (PMC11515759; doi:10.1186/s40337-024-01124-7)
Supplement: Supplementary file 1 — Supplementary Material 1 [file 40337_2024_1124_MOESM1_ESM.docx]

**INTERVIEW GUIDE FOR ADOLESCENT AND YOUNG ADULT PROVIDERS**

*Thank you so much for making the time to do this interview with me; I’m so grateful to be able to tap your expertise and really appreciate you taking the time to make this happen.*

*I’m going to go through a little bit of information about the interview before we start, OK?*

*My goal for this interview is to* ***learn*** ***from you*** *primarily about your experiences in working with ED patients, including those with Medi-Cal insurance, and understand how this may have changed in the context of FBT program starting up in SF, as well as the pandemic.*

*As part of this study, you agreed for us to record this interview, which will allow me to listen rather than spending time taking notes.* ***Are you okay with me starting the recording now?***

*If you’re uncomfortable answering any question, you’re free not to answer. You are also free to stop the interview and change your mind about participating at any time. I anticipate the interview will take about an hour. Do you have any questions before we get started?*

***Note: Interview questions are organized by CFIR domains (the intervention, inner and outer setting, the individuals involved, and the process by which implementation is accomplished).

**OVERVIEW**

**To start, how did you come to work with patients with eating disorders?**

Anything in particular that has made you feel more or less interested in learning more about eating disorders?

**Tell me generally about your experiences in treating patients with eating disorders.**

- *What* about treating patients with eating disorders has been different or challenging for you, compared to your work with other adolescents?
- *What* about treating patients with eating disorders has been easier for you?

Tell me about how COVID-19 has impacted your patient care.

**Turning now to your work eating disorder patients who are insured by Medi-Cal, I’m interested to hear about your experiences treating these cases and navigating the publicly-funded mental health service system.**

- What are some the challenges that you encounter in trying to help your patients access mental health care in county mental health systems?

How does your work differ, if at all, between patients who are covered by private insurance compared to those covered by Medi-Cal?

**How is your role as a provider different, if at all, for patients who will or are receiving FBT versus those who are receiving another kind of treatment?**

**What kind of changes do you think are needed to improve Medi-Cal services for youth with eating disorders across other counties?**

**CHARACTERISTICS OF THE INTERVENTION**

**What was your initial impression about FBT when you first learned about it?**

- What is your impression of FBT for youth with AN or Atypical AN who are covered by Medi-Cal?
- What challenges did you observe coming up for patients who are insured by Medi-Cal, or difficulties you have had you had in supporting their treatment?
  - Did you make any adaptations to your work to support these patients differently?

**How effective do you think FBT can be for patients with Medi-Cal?**

- About how many patients with Medi-Cal have you treated who accessed FBT? How effective do you think FBT has actually been for these patients?
  - How has the pandemic impacted how you feel about FBT or best treatment options?
  - Has the impact of the pandemic been any different for the publicly-insured patients that you treat?

**IMPLEMENTATION PROCESS**

**How did collaboration with FBT providers in SF occur? How effective was the process? What either helped or challenged you in collaborating with new providers?**

- What was your experience of co-managing care with providers in SF County compared to other counties?
- How much did you feel you could trust the FBT clinicians to manage your ED patients?

**What didn’t I ask you about supporting youth in FBT that is an important part of your experience?**

**INNER AND OUTER SETTING**

*Now I want to shift to asking more about your environment at work, and the health care context for the patients with Medi-Cal that you serve, and how this environment may have influenced care.*

**What resources did you feel you needed to manage patients with eating disorders who are covered by Medi-Cal? How much work was it for you to access these resources, and how supported did you feel in doing so?**

**What’s your sense of how well the system re-organized to facilitate San Francisco Medi-Cal referrals to FBT?**

**What external or systems-level factors made it challenging to support FBT?** (follow to ask specifically about medical/mental health integration and other systems issues, case management, supervision, peers)

**Overall, how well supported do you think families felt who were receiving FBT through the county?**

*We’ve reached the end of the interview. I’m so grateful to you for spending this time with me to learn more about your experiences. Is there anything else that would be relevant to share about your experiences in providing care to young people with eating disorders that I didn’t ask you about? Do you have any questions about anything we discussed today?*
